# The waiting room of uncertainty: digital patient support for potentially bad news—a scoping review

**DOI:** 10.3389/fdgth.2025.1706839

**Published:** 2025-11-19

**Authors:** Martina Kellermann, Charlotte Wetterauer, Gerd A. Kullak-Ublick, Marcus Cheetham

**Affiliations:** 1 Department of Internal Medicine, University Hospital Zurich, Zurich, Switzerland; 2 Department of Clinical Ethics, University Hospital of Basel (USB), Basel, Switzerland; 3 University Psychiatric Clinics Basel (UPK), Basel, Switzerland; 4 University Children's Hospital Basel (UKBB), Basel, Switzerland; 5 Geriatric University Medicine FELIX PLATTER Basel (UAFP), Basel, Switzerland; 6 Department of Clinical Pharmacology and Toxicology, University Hospital Zurich, Zurich, Switzerland

**Keywords:** receiving bad news, breaking bad news, oncology, patient aids, digital health technology, shared decision making, patient preferences

## Abstract

*Breaking bad news* (*BBN*) of serious or life-threatening diagnoses is common in oncology and often induces significant patient anxiety and distress. The anticipation of such news can also cause considerable distress, prompting patients to adopt proactive coping strategies, such as information seeking, while waiting for the news. Although the use of traditional and emerging digital technologies to assist patients across diverse aspects of cancer care has grown considerably, their role in assisting patients while they await the possibility of *receiving bad news* (*RBN*) remains unclear. We conducted a scoping review, following PRISMA-ScR guidelines, to identify studies on digital interventions, at any stage of realization, that aim to aid patient preparation for potential bad news, to map characteristics of these interventions (e.g., target diagnoses, design features) and to assess reported outcomes from usability to implementation. Using broad search terms related to digital technology, patient support and RBN and/or BBN in oncology across many databases, we identified 1020 articles. Most of these targeted healthcare professionals and BBN training (delivered with digital technology) and some referenced digital technology in the context of patients and bad news. However, none of the 22 articles that reached full text screening addressed any form of digital support for patients in relation to RBN. These findings highlight a striking gap in the use of digital support for this personally significant aspect of the patient journey. We contextualize this gap by discussing the value of digital support for RBN from a patient perspective, addressing why the news waiting period has received no attention in the BBN literature and highlighting the conceptual and practical intersections between RBN and *shared decision-making* (*SDM*), including consideration of common and distinct design features of patients aids for RBN and for SDM. Finally, we outline future research directions to address this significant and unmet need.

## Introduction

*Breaking bad news* (*BBN*) is a challenging event in oncology, occurring when patients are informed of a serious or life-threatening diagnosis or an adverse progression of existing cancer (1, 2). BBN can place high demands on doctors and trigger considerable anxiety and distress in patients (3, 4). The effectiveness of patient-doctor communication at this moment can shape patients' emotional responses, coping strategies, satisfaction with care and clinical outcomes (3, 5–9). In view of this, considerable research and training has focused on enhancing clinicians' BBN skills (9–11).

Research on patient experience has primarily focused on the moment at which bad news is disclosed, with particular attention to patients' cognitive, behavioural and emotional reactions to the news [for a corresponding definition of BBN, see (12)]. However, some of this research indicates substantial discrepancies between how bad news is delivered and how patients prefer *receiving bad news (RBN)* (13–16). Even though the burden on patients and their families while waiting for news can be considerable [e.g., (17, 18)], studies of patient experience and their preferences during the news waiting period and in preparation for the possibility of RBN remain scarce [e.g., (13, 19–21)].

The medical news waiting period refers to the interval of psychological and clinical uncertainty between initial suspicion or indication of a serious or life-threatening condition and the clinical communication of bad news. This period can occur at different moments during the patient journey (22), such as when waiting for screening or diagnostic test results, assessments of therapy effectiveness, findings of disease recurrence during ongoing surveillance, and decisions about transitioning to palliative care (22). Depending on the clinical context, cancer type and healthcare setting, the news waiting period can often span weeks to months (23). While the subjective perception of what counts as bad news may vary, and the actual and perceived duration of the news waiting period may deviate (24–24), patients often experience intense uncertainty, anxiety, fear and distress during this period (25–28).

Rather than passively enduring this uncertainty, cancer patients often adopt various emotional and behavioural coping strategies, such as searching for information online, seeking reassurance from family or peers, managing expectations, and preparing questions for clinicians (29–33). While these efforts to prepare in some way for the possibility of RBN may provide some relief (29, 34–37), they can also expose patients to misleading or irrelevant information, increase anxiety, undermine coping and risk maladaptive responses such as avoidance or catastrophizing (38–40). Waiting for potentially bad news can be more distressing than RBN itself (32, 41, 42), evoking clinical levels of anxiety and depression (27, 28, 43) and elevated cortisol levels comparable to those in cancer patients (44).

Purpose-designed supportive interventions have the potential to provide structured, evidence-based assistance tailored to patients' specific needs. Such interventions might enhance patient preparation for the possibility of RBN by delivering relevant, adequate and comprehensible patient-centered information about potential clinical outcomes, offering context-specific explanations and clarifications, correcting common misconceptions, facilitating adaptive emotional and behavioural coping strategies, and helping to enhance patient communication during clinical encounters (45–47). By incorporating such features, well-designed digital patient support tools could significantly strengthen patients' experience of feeling informed, supported and prepared while awaiting news.

Traditional and emerging digital technologies are increasingly deployed in oncology to provide remote patient support across the cancer care continuum. These include patient aids for predicting and assessing cancer risk (48, 49), supporting diagnostics and screening (30, 50–52), connecting patients with providers (53), providing care instructions and educational information (54–56), supporting shared decision making (SDM) (57), supporting cancer care, chemotherapy- and radiation therapy-related symptom monitoring and management (58), supporting cancer pain management (59, 60), offering emotional support and mental health tracking (61–63), addressing patients' needs and concerns (64), and aiding psychosocial intervention for post-treatment cancer survivors (65).

However, it remains uncertain whether purpose-designed digital support specifically targeting patients anticipating and awaiting the possibility of RBN is currently available or under development. None of the recent reviews of digital technology in oncology shed light on this (e.g., (66–70). The burden of waiting for potentially bad news coupled with the prevalence of proactive coping strategies during this period (32, 41, 42, 71, 72) points to a potentially unmet need in these patients. Rapid developments in conversational AI, such as ChatGPT, and the growing number of studies evaluating their use as patient-friendly resources for cancer knowledge, management and emotional support (73–75) suggest that digital tools could potentially be used to address these unmet needs.

The aim of this scoping review was to systematically map existing purpose-designed digital interventions to support oncology patients awaiting the possibility of RBN, and, where gaps exist, to conceptualize future directions for intervention development. Our research question focused on identifying these interventions, their key design features and how they have been evaluated. To this end, we sought studies reporting interventions at any stage of realization (i.e., conceptualization, design, development, evaluation, implementation) for any group of oncology patients awaiting the possibility of RBN. We aimed to characterize the identified interventions in terms of their specific features, including target users and diagnoses, timing of use within the patient journey, and design, and the dimensions of their evaluation such as usability, acceptability, user satisfaction, accessibility, implementation, and effectiveness in improving patient outcomes (e.g., experience of RBN, anxiety, patient–doctor communication, patient–doctor relationship before, during and after RBN).

## Methods

We conducted a scoping review to systematically search and synthesize studies that have reported the use of digital technology to support patients in relation to RBN and BBN, without aiming to appraise the methodological quality of these studies critically (76, 77). The scoping review has five stages, comprising determination of the research question, identification of relevant studies, selection of relevant studies, charting of the data, and collection, summarization and reporting of results (78). We conducted this review according to the PRISMA-ScR (Preferred Reporting Items for Systematic Reviews and Meta-Analyses extension for Scoping Reviews) checklist (79). Ethics approval was not required for the purpose of this scoping review.

### Eligibility criteria, data sources and search strategy

We covered the topics of *digital technology* for the purpose of *patient support for RBN and/or BBN* in the domain of *oncology*. We considered the possibility that digital *support for RBN and/or BBN* might have features in common with the design, development and content of *Patient Decision Aids* (*PtDAs*) for SDM (e.g., information about disease, diagnosis, treatment, risks and uncertainties, what to expect during or after a consultation) (80). For these reasons, we added SDM to the search in case this returned any articles that address digital support for RBN and/or BBN in the context of SDM and PtDAs. The search was limited to articles published in English, with no limitation on the time of publication.

A qualitative systematic literature search of publications was conducted in eight electronic databases: MEDLINE (Ovid), EMBASE (Ovid), Web of Science Core Collection, CINAHL, Cochrane Library (CENTRAL), IEEE Xplore Digital Library, Psych Index and Google Scholar. We defined the time of publication as the period between database inception and the time of the search (August 2025). Every individual search MeSH term was supplemented with relevant free text terms and, where appropriate, the free text terms were truncated to include alternative word endings (see Supplementary Table 1, for information on the search strategy). We also searched ClinicalTrials.gov for on-going trials and ProQuest for unpublished dissertations about digital technology in relation to BBN and RBN (81, 82).

### Study selection

Following the search of publications in the databases, we applied a two-step study selection process. Before this process, two reviewers screened a sample of the same 30 records to ensure consistency in the application of the procedure. In the first step of the process, these two reviewers manually and independently screened study titles and abstracts for inclusion criteria and categorized each study as to be *included*, *not included* or as *unclear* for inclusion in the next step of the study selection process (i.e., full article screening). Inter-rater reliability was assessed for this first step, before disagreements or uncertainties were discussed or resolved. A third reviewer was available to adjudicate unresolved cases.

If screening of titles and abstracts indicated that the article was not to be included in the next step of the selection process, each reviewer provided the reason for exclusion. To this end, each reviewer categorized each article according to whether the following descriptors applied in the following order: “healthcare professionals are the exclusive target group of interest of the article”, “article does not consider a medical issue”, “article is not about oncology“, “article does not consider digital technology“, “article does not consider BBN or RBN” and “other reasons for exclusion (e.g., books, conference papers, magazines, policy reports, funding related, workshops, symposiums, lectures).” The terms BBN and RBN were used as referring to any form of bad news. In this stepwise exclusion procedure, one criterion was sufficient to trigger the exclusion of an article.

The categorization of a record by one or both reviewers as being included or unclear lead to the automatic inclusion of the study in the next step. Both reviewers conducted full article screening of all studies that reached this step, separately. The final selection of studies was determined by both reviewers, critically discussing each study that resulted from the full article-screening step.

### Data charting process

**Figure 1:**
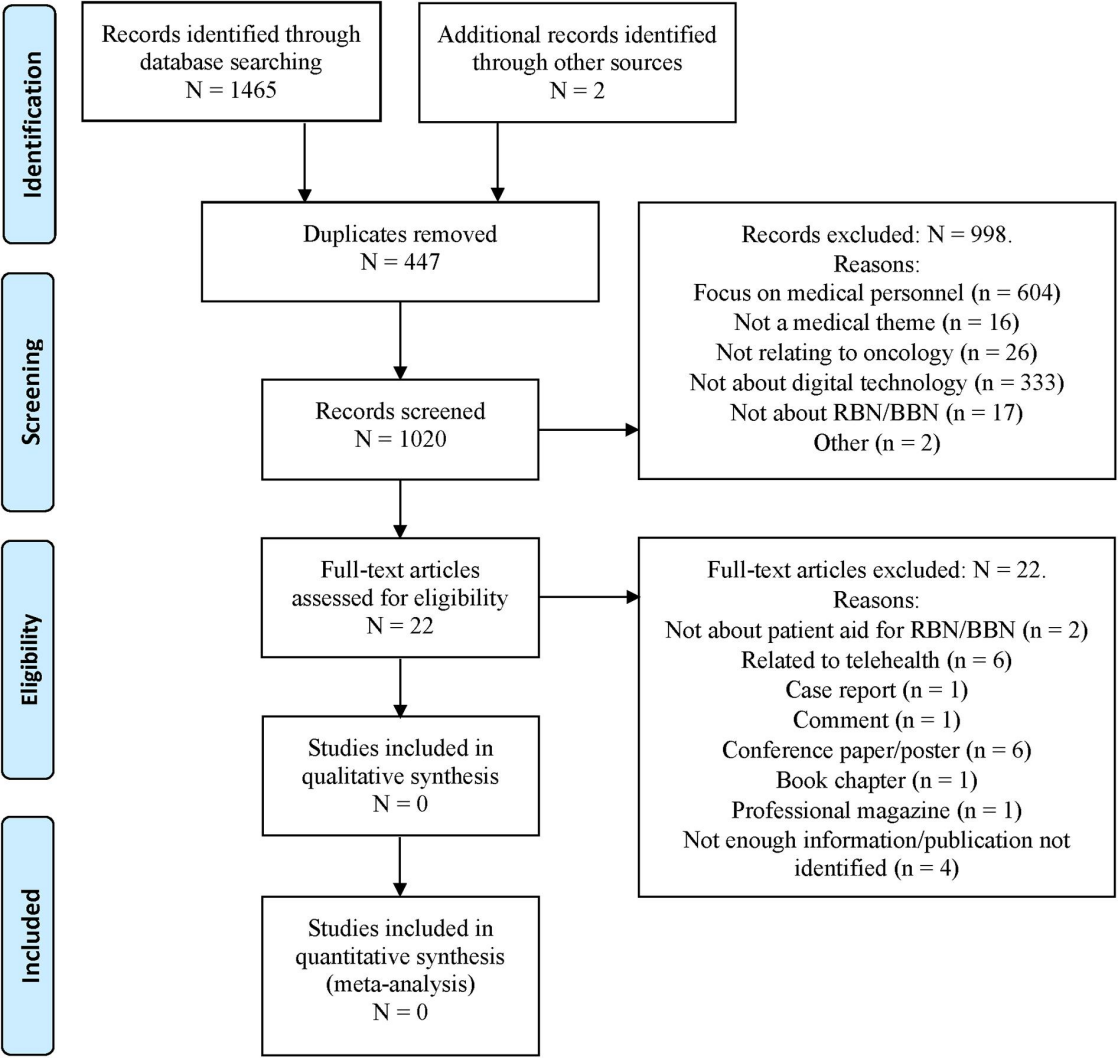
PRISMA-ScR (preferred reporting items for systematic reviews and meta-analyses extension for scoping reviews) flow diagram generated using distillerSR, showing the study selection process including the records that were identified and screened (after removal of duplicates), excluded during the manual screening process, remaining as full-text articles, and excluded after full text screening.

EndNote™ was used to manage the studies that were identified and retrieved from the database searches (83). DistillerSR review software (Evidence Partners) was used to support and document the two-step study selection process of studies (see Figure 1, for the PRISMA-ScR flow diagram) (84).

## Results

### Study screening

The first step of the study selection process returned 1,020 articles. Two reviewers manually screened these, separately. There was strong consistency in screening, with a Cohen's *κ* of 0.93 and a mean percentage agreement of 97.84% (*SD* = 0.01%) for paper exclusion, indicating minimal deviation between reviewers (85). This result likely reflects the clarity and specificity of the inclusion criteria (i.e., digital support for patients for BBN/RBN in oncology) and the sparsity of studies meeting these criteria, which rendered the distinction between eligible and ineligible records largely unambiguous.

Based on the stepwise exclusion procedure, 998 articles were excluded (see Figure 1). Of these, 604 articles were found to focus exclusively on healthcare professionals, 16 did not relate to medicine, 26 did not relate to oncology, 333 made no reference to digital technology, 17 made no mention of RBN or BBN, and 2 publications were excluded because the abstract contained no information. This procedure resulted in 22 articles reaching full-text screening in the second step of the study selection process. After full text screening, we excluded all 22 of these articles because they failed to meet any of the inclusion criteria (see Supplementary Table 2 for a summary of excluded articles and reasons for exclusion as well as Figure 1 for these reasons).

## Discussion

Given that digital tools and technologies are otherwise used to support cancer patients across the care continuum (30, 53, 56, 61, 64) and that cancer patients report unmet needs and preferences in relation to RBN (13), it is reasonable to ask whether any digital solutions could also provide technically feasible and medically practicable support for patients awaiting the possibility of RBN.

We conducted a comprehensive scoping review for articles on any digital tools at any stage of realization (i.e., conceptualization, design, development, evaluation, implementation) for use by any patients in any relation to RBN in oncology. The search strategy combined multiple databases and broad search terms to maximise coverage of relevant literature, including clinical trials and unpublished dissertations. After screening a substantial number of potentially relevant articles, we found no evidence in these databases of digital patient aids for use while waiting for news.

In view of this striking gap, we explore (1) why these patient aids warrant attention, (2) why the BBN literature may have overlooked the use of digital tools in the news waiting period, and (3) key directions for future research and development of these tools.

### Why digital patient aids for RBN warrant attention

The literature documents that cancer patients experience considerable uncertainty while waiting for potentially unfavourable results of diagnostic tests and procedures (43). This uncertainty relates to difficulties making sense of the meaning, severity and impact of the illness to which the potential news might or does relate (29, 32). These difficulties are associated with factors such as insufficient, incomplete, or conflicting information (i.e., information ambiguity), information that is difficult to comprehend (i.e., information complexity), constraints of limited time, knowledge and mental processing capabilities on processing information (i.e., information processing capacity) and the challenges of evaluating the likelihood of a negative result (i.e., risk evaluation) (29, 86). A range of factors can influence uncertainty, such as differences in the dispositional tendency to cope with uncertainty, in the subjective perception of what counts as bad news, and the actual and perceived duration of the news waiting period (24, 87).

Uncertainty in the news waiting period can provoke intense and sometimes overwhelming negative affect, including worry, anxiety, distress and fear (25–28). The perceived threat to personal well-being or survival of a pending diagnosis can elicit various responses, including affective and cognitive coping strategies, such as maintaining optimism, engaging in distraction, suppressing negative affect, seeking emotional support of others, and reappraising or reframing uncertainty (32, 33). Typically, patients also seek information online and via friends and acquaintances (sometimes about comparative cases known to them) to gain clarity and reduce uncertainty (29, 34–37).

Online information seeking during the news waiting period can help cancer patients fulfil information needs (71). These needs may arise when other patient support resources are lacking, before speaking with a clinician, when the clinician's consultation time is limited, or when patients feel under-informed (i.e., information insufficiency) or overwhelmed with the information they receive at consultation (88–90). Information needs can reflect the patient's recognition of a knowledge gap that must be bridged to make sense of their situation (though not all patients recognize a knowledge gap or pursue information even when a gap is recognized) (91, 92). Seeking information about a possible diagnosis and its meaning for a patient can reassure some patients, help to give patients a sense of some control over how much or little information they process at a given time, help them form a picture of what to expect at the consultation, feel better prepared for and able to participate in the consultation and form a picture of what might happen after RBN (45, 80).

#### Implications

The main implication of the preceding evidence is that there is a significant opportunity to meet unmet patient needs during the news waiting period by means of digitally-mediated supportive interventions. These interventions would require carefully designed digital tools and resources that are accessible, relevant, timely, accurate and trustworthy may help patients and their companions (93–96). Without this support, patients and companions might encounter unreliable, irrelevant, misleading or (emotionally and cognitively) overwhelming information that could exacerbate anxiety and distress, and undermine effective coping, daily functioning and ability to more effectively adjust to potentially distressing information while waiting for news and in the clinical encounter (38–40).

### Why the BBN literature may have overlooked the news waiting period

Given the growing use of digital support tools for oncology patients (30, 53, 56, 61, 64), the question arises as to why digital tools for patients awaiting potentially bad news has received no attention (80). Four possible reasons for this are explored in the following.

#### Exclusive focus on those consultations where bad news is delivered

The focus on consultations in which BBN is delivered implicitly excludes those consultations in which BBN could occur but does not (12, 97). For patients awaiting potentially bad news, the distinction between consultations with and without bad news is likely to be irrelevant. The challenges of BBN are of course reflected in the enduring emphasis on developing BBN guidelines, tools and training to improve communication when delivering bad news (2, 12, 20, 97–106). Consistent with this, the manually screened articles in this review related largely to communication training for healthcare professionals, many of which reporting the design, implementation and evaluation of digital tools to aid BBN training. In contrast, a negligible number of articles addressed patients' needs, preferences and experiences of BBN or RBN and no articles considered digital tools to aid patients.

#### BBN as a single point-in-time event

The BBN literature tends to focus on a single discrete, or isolated, piece of bad news (e.g., a new and unexpected cancer diagnosis) disclosed at a given time point (6, 107). However, from a patient perspective, bad news can be part of an ongoing process (108) that can span between an initial consultation (when this process begins) and the news consultation (109). In some cases, bad news is already implicit before the initial consultation (e.g., first symptoms where there is a known or suspected cancer risk) (110). In other words, news disclosure can also be viewed as an unfolding process rather than a single event, highlighting the importance of considering patient experiences throughout the news waiting period. This is also consistent with the clinical experience of doctors and nurses (111, 112) who report that BBN can entail an incremental process of news disclosure across several patient consultations or interactions (113–116) in a process that is interlaced with many other pieces of information about diverse aspects of the patient's care (117).

#### The unilateral delivery of bad news

The BBN literature tends to focus on the delivery of bad news from doctor to patient (2, 6, 20, 100). The preceding literature on patient experience of RBN highlights that the flow of information is not always unilateral and could be understood as a bilateral process of information sharing during the receiving and breaking of bad news [e.g. (108–110)]. This understanding has already been advocated as part of a conceptual shift away from a unilateral perspective in BBN to a joint perspective of information exchange between patient and doctor (6). Consistent with this conceptual shift, patients report a need and preference for support that facilitates participation in a shared approach to processing information about bad news and to making decisions with their doctor (6, 13, 118).

#### Patients as the only recipients of bad news

The BBN literature tends to focus on the consultation as a clinician-patient dyad. However, the clinical reality is that companions' emotional and cognitive support plays a critical role in the news waiting period and that patients often prefer to have a personal companion with them during news disclosure (20, 102, 106, 119) who can share the distressing burden of bad news (21, 120–122). Companions also provide cognitive support during disclosure, sometimes facilitating a faster flow of information between those present at the consultation, raising important issues, asking significantly more questions, and recalling more information after the consultation (114, 117, 123, 124).

#### Implications

Based on the preceding literature and articles screened in this review, it appears that the predominant conceptualization of bad news in the prevailing BBN-centered framework does not readily accommodate consideration of the news waiting period, even though this period appears to constitute a critical stage of the patient journey (32). Augmenting this framework with a complementary patient-centered framework for RBN and the news waiting period might pave the way to the development of suitable digital tools to support patient.

### Key directions for future research and development of digital interventions

The scarcity of relevant research on RBN and the news waiting period as well as the absence of literature on related digital tools hinders an evaluation of the potential feasibility, effectiveness and uptake of dedicated patient aids for RBN. Several avenues of research are needed to enable this evaluation and development of these aids.

#### Needs assessment

A foundational step in developing effective patient aids for RBN is a comprehensive needs assessment to understand whether, when and for whom patient aids for RBN might be helpful and practicable. This includes identifying factors that might help or hinder the development, adoption and effectiveness of these aids. This assessment requires rigorous qualitative and quantitative research coupled with a user-centered design approach (125–127). Key components include:

- Current patient strategies: Examine how patients prepare for potential bad news without the support of dedicated aids, focusing on experiences (e.g., uncertainty, anxiety) and behaviours (e.g., coping, information-seeking) in various clinical settings and at different time points.
- Patient preferences: Explore the format, type and focus of supportive interventions, considering diverse populations and their unique needs.
- Contextual factors, facilitators, and barriers: Identify suitable clinical contexts and consider patient, clinician, organizational and technological perspectives, as well as facilitators (e.g., access, reduced burden) and barriers (e.g., digital literacy, resistance) to successful implementation.

Multi-stakeholder engagement with patients, companions and healthcare providers are to conducting this assessment and to subsequently guiding the design, development and implementation of user-centered aids to ensure that these are sufficiently supportive of end users' real-world needs.

#### Design of patient aids for RBN

Assuming needs assessments favour the development of patient aids for RBN [in a user-centered design approach (128, 129)], their development might entail the following key components:

- Supportive interventions: Depending on patient needs and preferences, supportive interventions might include, for example, (adequate, relevant, accurate, credible, trustworthy and usable) educational content about possible news outcomes, explanations and clarifications tailored to the clinical context, information to correct common misconceptions, guided coping strategies tailored self-reported stress levels, on-demand access and just-in-time support [e.g., before results are posted in a patient portal (130, 131)].
- Design features: Suitably designed digital patient aids for RBN could facilitate the delivery of these supportive interventions. The design features of these aids might include, for example, adaptive personalized support to accommodate different patient preferences, media formats for information delivery, digital health and reading literacy levels, and cultural contexts, with multi-language options. Features like these might promote acceptance, engagement and sustained use (46, 47).
- Effectiveness: Digital patient aids for RBN should be effective in supporting patients while awaiting news. Besides outcomes like reduced anxiety and distress while waiting, research should also assess the downstream impact of this support on outcomes such as the quality of patient–clinician communication, clinician satisfaction, patient satisfaction and trust, and treatment adherence (132, 133).

#### Conceptualization

Consolidating supportive interventions under the umbrella of Patients Aids for RBN contributes to a coherent, scalable and patient-centered framework for addressing the psychological challenges of waiting for bad news. Research and development should be guided by feasibility, practicability, and ethical considerations, with particular emphasis on recognizing patient agency. Crucially, such aids reposition the patient's role from a largely passive recipient of bad news within the consultation, as assumed in the prevailing BBN framework, to a stronger and earlier active role in navigating uncertainty during the waiting period (111, 134).

Developing digital patient aids for RBN could be considered in the context of PtDAs for SDM. While PtDAs often address potential diagnoses (e.g., screening for cancer) that might lead to RBN in the future, RBN (e.g., about a negative change in an existing cancer) can also precede the use of a PtDA (e.g., about further treatment) (80). In other words, the conceptual focus of patient aids for RBN and of PtDAs is closely related in the care continuum but they serve distinct purposes. In the absence of literature on digital support for RBN, future work toward the conceptualization, design, development, implementation and evaluation of patient aids for RBN might benefit from the wealth of knowledge that has accrued over the last 30 years on the design and development of PtDAs for SDM (135).

### Practical implications

Given the absence of existing interventions or literature on digital patient aids for the news waiting period), these findings highlight a critical opportunity for clinicians, developers and policymakers to pioneer this area of support. Clinicians could acknowledge the psychological burden faced by patients during this waiting period and advocate for the development of tools that provide clear, supportive and accessible guidance. Developers are encouraged to design and pilot digital aids that help patients manage uncertainty, building on frameworks from related fields like PtDAs. Policymakers should prioritize funding and policies that foster ethical, feasible and scalable innovation in this domain. Early multidisciplinary collaboration and rigorous evaluation will be crucial to ensure that such interventions effectively address patient needs and successfully integrate into oncology care.

### Limitations

A limitation of scoping reviews can be that they provide a broad view of the relevant body of literature and generate similarly broad findings without providing great insight and depth into the area of enquiry. Nevertheless, this approach enabled the identification of a potentially significant gap that might have otherwise continued to escape attention. Given the rapid growth in digital technology for remote aiding of cancer patients and the keen development of PtDAs for SDM, this result was not expected. While we used a broad range of databases and search terms to maximise coverage of terms relating to digital technology, patient support and RBN and/or BBN in oncology, we cannot exclude the possibility that this review may have missed relevant studies. In addition, our review was limited to English-language publications, potentially excluding work in other languages [e.g. (80),]. The use of Google Scholar, while useful for capturing grey literature, is limited in terms of search replicability and precision.

Notably, while preparing this manuscript, we identified a new conversational AI-supported tool that was first reported in a conference abstract (136). This tool provides personalized, proactive support and guidance to cancer patients, though it is not clear if this extends to those waiting for potentially bad news (137). Similarly, a recent conceptual contribution outlines the design and rationale of a trauma-sensitive, web-based platform (the Virtual Waiting Room*)* to address the emotional and informational needs of patients during the news waiting period (138). Together, these examples signal the arrival of a new wave of digital interventions that could support patients in the waiting period. Future research should critically evaluate such emerging tools to ensure they are effective in addressing the complex realities of patients' experiences in the waiting period. This scoping review is therefore timely, as it highlights a critical gap and identifies a promising future area for digital intervention development.

### Conclusions

This review highlights a striking gap in the literature on digital interventions for patients in oncology: Even though waiting for the possibility of receiving serious or life-threatening news can be highly distressing and elicit a range of proactive coping strategies over variously long periods of time, the opportunity to apply digital tools to support these patients has not been explored. Given the potential benefits of such tools, the prevailing clinician-centered approach to news with its focus on the moment of bad news disclosure could be augmented with a complementary patient-centered approach that embraces the potential of digital technologies to aid patients who are waiting for potentially bad news. Digital tools designed to empower patients during the waiting period hold promise for alleviating anxiety, enhancing health literacy, and fostering active participation in subsequent consultations. Assuming that patient aids for RBN are feasible and practicable, realizing these requires a concerted effort to conceptualize, design, develop, evaluate and implement them. Traditional and emerging digital technologies are poised to play a decisive role in bridging this gap.

## Data Availability

The original contributions presented in the study are included in the article/Supplementary Material, further inquiries can be directed to the corresponding author.

## References

[1] Fallowfield L Jenkins V. Communicating sad, bad, and difficult news in medicine. Lancet. (2004) 363(9405):312–9. 10.1016/S0140-6736(03)15392-5 14751707

[2] Baile WF Buckman R Lenzi R Glober G Beale EA Kudelka AP. SPIKES-A six-step protocol for delivering bad news: application to the patient with cancer. Oncologist. (2000) 5(4):302–11. 10.1634/theoncologist.5-4-302 10964998

[3] Gattellari M Butow PN Tattersall MH Dunn SM MacLeod CA. Misunderstanding in cancer patients: why shoot the messenger? Ann Oncol. (1999) 10(1):39–46. 10.1023/A:1008336415362 10076720

[4] Kagawa-Singer M. Teaching Culturally Competent Communication with Diverse Ethnic Patients and Families. New Challenges in Communication with Cancer Patients. Vol. 1. New York, NY: Springer (2012). p. 365–75.

[5] Fallowfield L Jenkins V. Effective communication skills are the key to good cancer care. Eur J Cancer. (1999) 35(11):1592–7. 10.1016/S0959-8049(99)00212-9 10673967

[6] Berger JT Miller DR. Physicians should stop breaking bad news. J Gen Intern Med. (2022) 37(13):3475–6. 10.1007/s11606-022-07566-6 35411537 PMC9551006

[7] Choudhry A Hong J Chong K Jiang B Hartman R Chu E Patients’ preferences for biopsy result notification in an era of electronic messaging methods. JAMA Dermatol. (2015) 151(5):513–21. 10.1001/jamadermatol.2014.5634 25831475

[8] Kagawa-Singer M. Teaching culturally competent communication with diverse ethnic patients and families. New Challeng Commun Cancer Patients. (2013):365–75. 10.1007/978-1-4614-3369-9_30

[9] Abel J Dennison S Senior-Smith G Dolley T Lovett J Cassidy S. Breaking bad news—development of a hospital-based training workshop. Lancet Oncol. (2001) 2(6):380–4. 10.1016/S1470-2045(00)00393-4 11905755

[10] Back AL Arnold RM Baile WF Fryer-Edwards KA Alexander SC Barley GE Efficacy of communication skills training for giving bad news and discussing transitions to palliative care. Arch Intern Med. (2007) 167(5):453–60. 10.1001/archinte.167.5.453 17353492

[11] Hawken S. Strategies for dealing with the challenging patient. New Zealand Family Physician. (2005) 32(4):266.

[12] Ptacek JT Eberhardt TL. Breaking bad news. A review of the literature. JAMA. (1996) 276(6):496–502. 10.1001/jama.1996.03540060072041 8691562

[13] Seifart C Hofmann M Bär T Knorrenschild JR Seifart U Rief W. Breaking bad news–what patients want and what they get: evaluating the SPIKES protocol in Germany. Ann Oncol. (2014) 25(3):707–11. 10.1093/annonc/mdt582 24504443 PMC4433514

[14] Grimes GC Reis MD Budati G Gupta M Forjuoh SN. Patient preferences and physician practices for laboratory test results notification. J Am Board Fam Med. (2009) 22(6):670–6. 10.3122/jabfm.2009.06.090078 19897696

[15] Schofield MJ Sanson-Fisher R Halpin S Redman S. Notification and follow-up of pap test results: current practice and women’s preferences. Prev Med. (1994) 23(3):276–83. 10.1006/pmed.1994.1039 8078847

[16] Peres M Wellman M. Notification of papanicolaou smear results: a survey of women’s experiences and preferred means of notification. Australian New Zealand J Obstet Gynaecol. (2001) 41(1):82–5. 10.1111/j.1479-828X.2001.tb01300.x 11284654

[17] Awsare N Green J Aldwinckle B Hanbury D Boustead G McNicholas T. The measurement of psychological distress in men being investigated for the presence of prostate cancer. Prostate Cancer Prostatic Dis. (2008) 11(4):384–9. 10.1038/pcan.2008.21 18427569

[18] Liao M-N Chen M-F Chen S-C Chen P-L. Uncertainty and anxiety during the diagnostic period for women with suspected breast cancer. Cancer Nurs. (2008) 31(4):274–83. 10.1097/01.NCC.0000305744.64452.fe 18600114

[19] Peteet JR Abrams HE Ross DM Stearns NM. Presenting a diagnosis of cancer—patients views. J Fam Practice. (1991) 32(6):577–81. 2040882

[20] Girgis A Sanson-Fisher RW. Breaking bad news: consensus guidelines for medical practitioners. J Clin Oncol. (1995) 13(9):2449–56. 10.1200/JCO.1995.13.9.2449 7666105

[21] Butow PN Kazemi JN Beeney LJ Griffin AM Dunn SM Tattersall MHN. When the diagnosis is cancer—patient communication experiences and preferences. Cancer-Am Cancer Soc. (1996) 77(12):2630–7. 10.1002/(SICI)1097-0142(19960615)77:12<2630::AID-CNCR29>3.0.CO;2-S 8640715

[22] Kreitler S. The phases of the confrontation with cancer. In: Psycho-Oncology for the Clinician: the Patient Behind the Disease. Cham: Springer (2019). p. 25–43.

[23] Gitlin M McGarvey N Shivaprakash N Cong Z. Time duration and health care resource use during cancer diagnoses in the United States: a large claims database analysis. J Manag Care Spec Pharm. (2023) 29(6):659–70. 10.18553/jmcp.2023.29.6.659 37276034 PMC10388018

[24] Dooley MK Sweeny K Howell JL Reynolds CA. Perceptions of romantic partners’ responsiveness during a period of stressful uncertainty. J Pers Soc Psychol. (2018) 115(4):677–87. 10.1037/pspi0000134 30047761

[25] Boivin J Lancastle D. Medical waiting periods: imminence, emotions and coping. Womens Health (Lond. (2010) 6(1):59–69. 10.2217/WHE.09.79 20088730

[26] Hamilton JG Hutson SP Moser RP Kobrin SC Frohnmayer AE Alter BP Sources of uncertainty and their association with medical decision making: exploring mechanisms in fanconi Anemia. Ann Behav Med. (2013) 46(2):204–16. 10.1007/s12160-013-9507-5 23637072

[27] Lampic C Thurfjell E Bergh J Sjödén PO. Short- and long-term anxiety and depression in women recalled after breast cancer screening. Eur J Cancer. (2001) 37(4):463–9. 10.1016/S0959-8049(00)00426-3 11267855

[28] Pineault P. Breast cancer screening: women’s experiences of waiting for further testing. Oncol Nurs Forum. (2007) 34(4):847–53. 10.1188/07.ONF.847-853 17723985

[29] Mishel MH. Uncertainty in illness. Image J Nurs Sch. (1988) 20(4):225–32. 10.1111/j.1547-5069.1988.tb00082.x 3203947

[30] Ghosh S Bhatia S Bhatia A. Quro: Facilitating User Symptom Check Using a Personalised Chatbot—Oriented Dialogue System. (2018). 51-6 p. 30040682

[31] Sweeny K. On the experience of awaiting uncertain news. Curr Dir Psychol Sci. (2018) 27(4):281–5. 10.1177/0963721417754197

[32] Sweeny K Cavanaugh AG. Waiting is the hardest part: a model of uncertainty navigation in the context of health news. Health Psychol Rev. (2012) 6(2):147–64. 10.1080/17437199.2010.520112

[33] Howell JL Sweeny K. Is waiting bad for subjective health? J Behav Med. (2016) 39:652–64. 10.1007/s10865-016-9729-7 26969093

[34] Lagoe C Atkin D. Health anxiety in the digital age: an exploration of psychological determinants of online health information seeking. Comput Hum Behav. (2015) 52:484–91. 10.1016/j.chb.2015.06.003

[35] Fox S Duggan M. Health online 2013. Health. (2013) 2013:1–55.

[36] Jaks R Baumann I Juvalta S Dratva J. Parental digital health information seeking behavior in Switzerland: a cross-sectional study. BMC Public Health. (2019) 19:225. 10.1186/s12889-019-6524-8 30791927 PMC6385444

[37] Ybarra ML Suman M. Help seeking behavior and the internet: a national survey. Int J Med Inform. (2006) 75(1):29–41. 10.1016/j.ijmedinf.2005.07.029 16129659

[38] Lillie H Katz RA Carcioppolo N Giorgi EA Jensen JD. Cancer information overload across time: evidence from two longitudinal studies. Health Commun. (2023) 38(9):1878–86. 10.1080/10410236.2022.2038866 35172651 PMC9378766

[39] Nagao N Tsuchiya A Ando S Arita M Toyonaga T Miyawaki I. The psychosocial influences of waiting periods on patients undergoing endoscopic submucosal dissection. Gastroenterol Nurs. (2017) 40(5):373–9. 10.1097/SGA.0000000000000216 26987103 PMC5625967

[40] Bartley N Napier CE Butt Z Schlub TE Best MC Biesecker BB Cancer patient experience of uncertainty while waiting for genome sequencing results. Front Psychol. (2021) 12:647502. 10.3389/fpsyg.2021.647502 33967906 PMC8100530

[41] Sweeny K Falkenstein A. Is waiting the hardest part? Comparing the emotional experiences of awaiting and receiving bad news. Pers Soc Psychol Bull. (2015) 41(11):1551–9. 10.1177/0146167215601407 26338852

[42] Nosarti C Roberts JV Crayford T McKenzie K David AS. Early psychological adjustment in breast cancer patients: a prospective study. J Psychosom Res. (2002) 53(6):1123–30. 10.1016/S0022-3999(02)00350-1 12479995

[43] Poole K. The emergence of the ‘waiting game': a critical examination of the psychosocial issues in diagnosing breast cancer. J Adv Nurs. (1997) 25(2):273–81. 10.1046/j.1365-2648.1997.1997025273.x 9044000

[44] Lang EV Berbaum KS Lutgendorf SK. Large-core breast biopsy: abnormal salivary cortisol profiles associated with uncertainty of diagnosis. Radiology. (2009) 250(3):631–7. 10.1148/radiol.2503081087 19244038

[45] Meredith C Symonds P Webster L Lamont D Pyper E Gillis CR Information needs of cancer patients in west Scotland: cross sectional survey of patients’ views. Br Med J. (1996) 313(7059):724–6. 10.1136/bmj.313.7059.724 8819442 PMC2352093

[46] Yen P-Y Bakken S. Review of health information technology usability study methodologies. J Am Med Inform Assoc. (2012) 19(3):413–22. 10.1136/amiajnl-2010-000020 21828224 PMC3341772

[47] Witteman HO Dansokho SC Colquhoun H Coulter A Dugas M Fagerlin A User-centered design and the development of patient decision aids: protocol for a systematic review. Syst Rev. (2015) 4(1):11. 10.1186/2046-4053-4-11 25623074 PMC4328638

[48] Nazareth S Hayward L Simmons E Snir M Hatchell KE Rojahn S Hereditary cancer risk using a genetic chatbot before routine care visits. Obstet Gynecol. (2021) 138(6):860–70. 10.1097/AOG.0000000000004596 34735417 PMC8594498

[49] Markun S Scherz N Rosemann T Tandjung R Braun RP. Mobile teledermatology for skin cancer screening: a diagnostic accuracy study. Medicine (Baltimore). (2017) 96(10):e6278. 10.1097/MD.0000000000006278 28272243 PMC5348191

[50] Costantini L Del Riccio M Piccoli E Lavecchia V Corradini E Bonaccorsi G Use of digital technologies to support cancer screening in community health promotion interventions: scoping review. Health Promot Int. (2023) 38(1):daac189. 10.1093/heapro/daac189 36757345

[51] Ruco A Dossa F Tinmouth J Llovet D Jacobson J Kishibe T Social media and mHealth technology for cancer screening: systematic review and meta-analysis. J Med Internet Res. (2021) 23(7):e26759. 10.2196/26759 34328423 PMC8367160

[52] Pangti R Mathur J Chouhan V Kumar S Rajput L Shah S A machine learning-based, decision support, mobile phone application for diagnosis of common dermatological diseases. J Eur Acad Dermatol Venereol. (2021) 35(2):536–45. 10.1111/jdv.16967 32991767

[53] Rarhi K Bhattacharya A Mishra A Mandal K. Automated medical chatbot. SSRN J. (2017). 10.2139/ssrn.3090881

[54] Ciria-Suarez L Costas L Flix-Valle A Serra-Blasco M Medina JC Ochoa-Arnedo C. A digital cancer ecosystem to deliver health and psychosocial education as preventive intervention. Cancers (Basel). (2022) 14(15):3724. 10.3390/cancers14153724 35954388 PMC9367518

[55] Chetlen A Artrip R Drury B Arbaiza A Moore M. Novel use of chatbot technology to educate patients before breast biopsy. J Am Coll Radiol. (2019) 16(9):1305–8. 10.1016/j.jacr.2019.05.050 31492408

[56] Zauderer MG Gucalp A Epstein AS Seidman AD Caroline A Granovsky S Piloting IBM watson oncology within memorial sloan kettering’s regional network. J Clin Oncol. (2014) 32(15_suppl):e17653-e. 10.1200/jco.2014.32.15_suppl.e17653

[57] Hao Y Liu Z Riter RN Kalantari S. Advancing patient-centered shared decision-making with AI systems for older adult cancer patients. Proceedings of the 2024 CHI Conference on Human Factors in Computing Systems (2024).

[58] Basch E Deal AM Kris MG Scher HI Hudis CA Sabbatini P Symptom monitoring with patient-reported outcomes during routine cancer treatment: a randomized controlled trial. J Clin Oncol. (2016) 34(6):557. 10.1200/JCO.2015.63.0830 26644527 PMC4872028

[59] Marthick M McGregor D Alison J Cheema B Dhillon H Shaw T. Supportive care interventions for people with cancer assisted by digital technology: systematic review. J Med Internet Res. (2021) 23(10):e24722. 10.2196/24722 34714246 PMC8590193

[60] Zheng C Chen X Weng L Guo L Xu H Lin M Benefits of mobile apps for cancer pain management: systematic review. JMIR Mhealth Uhealth. (2020) 8(1):e17055. 10.2196/17055 32012088 PMC7005688

[61] Jeddi Z Bohr A. Remote patient monitoring using artificial intelligence. In: Bohr A, Memarzadeh K, editors. Artificial Intelligence in Healthcare. London: Elsevier (2020). p. 203–34.

[62] Triberti S Savioni L Sebri V Pravettoni G. Ehealth for improving quality of life in breast cancer patients: a systematic review. Cancer Treat Rev. (2019) 74:1–14. 10.1016/j.ctrv.2019.01.003 30658289

[63] Ma D Orner D Ghaly MM Parashar B Ames JW Chen WC Automated health chats for symptom management of head and neck cancer patients undergoing radiation therapy. Oral Oncol. (2021) 122:105551. 10.1016/j.oraloncology.2021.105551 34700280

[64] Piau A Crissey R Brechemier D Balardy L Nourhashemi F. A smartphone chatbot application to optimize monitoring of older patients with cancer. Int J Med Inform. (2019) 128:18–23. 10.1016/j.ijmedinf.2019.05.013 31160007

[65] Chan R Crichton M Crawford-Williams F Agbejule O Yu K Hart N The efficacy, challenges, and facilitators of telemedicine in post-treatment cancer survivorship care: an overview of systematic reviews. Ann Oncol. (2021) 32(12):1552–70. 10.1016/j.annonc.2021.09.001 34509615

[66] Chen D Avison K Alnassar S Huang RS Raman S. Medical accuracy of artificial intelligence chatbots in oncology: a scoping review. Oncologist. (2025) 30(4):oyaf038. 10.1093/oncolo/oyaf038 40285677 PMC12032582

[67] Lawson McLean A Hristidis V. Evidence-based analysis of AI chatbots in oncology patient education: implications for trust, perceived realness, and misinformation management. J Cancer Educ. (2025) 40:1–8. 10.1007/s13187-025-02592-4 39964607 PMC12310775

[68] Borkar S Chakole S Prasad R Bansod S. Revolutionizing oncology: a comprehensive review of digital health applications. Cureus. (2024) 16(4):e59203. 10.7759/cureus.59203 38807819 PMC11131437

[69] Tuominen L Leino-Kilpi H Poraharju J Cabutto D Carrion C Lehtiö L Interactive digital tools to support empowerment of people with cancer: a systematic literature review. Support Care Cancer. (2024) 32(6):396. 10.1007/s00520-024-08545-9 38816629 PMC11139693

[70] Goumas G Dardavesis TI Syrigos K Syrigos N Simou E. Chatbots in cancer applications, advantages and disadvantages: all that glitters is not gold. J Pers Med. (2024) 14(8):877. 10.3390/jpm14080877 39202068 PMC11355580

[71] Ziebland S Chapple A Dumelow C Evans D Prinjha S Rozmovits L. How the internet affects patients’ experience of cancer: a qualitative study. Br Med J. (2004) 328(7439):564-+. 10.1136/bmj.328.7439.564 15001506 PMC381051

[72] Lebel S Jakubovits G Rosberger Z Loiselle C Seguin C Cornaz C Waiting for a breast biopsy: psychosocial consequences and coping strategies. J Psychosom Res. (2003) 55(5):437–43. 10.1016/S0022-3999(03)00512-9 14581098

[73] Yeo YH Samaan JS Ng WH Ting P-S Trivedi H Vipani A Assessing the performance of ChatGPT in answering questions regarding cirrhosis and hepatocellular carcinoma. Clin Mol Hepatol. (2023) 29(3):721. 10.3350/cmh.2023.0089 36946005 PMC10366809

[74] Bilgin GB Bilgin C Childs DS Orme JJ Burkett BJ Packard AT Performance of ChatGPT-4 and bard chatbots in responding to common patient questions on prostate cancer 177Lu-PSMA-617 therapy. Front Oncol. (2024) 14:1386718. 10.3389/fonc.2024.1386718 39070149 PMC11272524

[75] Ye Z Zhang B Zhang K Méndez MJG Yan H Wu T An assessment of ChatGPT’s responses to frequently asked questions about cervical and breast cancer. BMC Women’s Health. (2024) 24(1):482. 10.1186/s12905-024-03320-8 39223612 PMC11367894

[76] Arksey H O'Malley L. Scoping studies: towards a methodological framework. Int J Soc Res Methodol. (2005) 8(1):19–32. 10.1080/1364557032000119616

[77] Levac D Colquhoun H O'Brien KK. Scoping studies: advancing the methodology. Implement Sci. (2010) 5:69. 10.1186/1748-5908-5-69 20854677 PMC2954944

[78] Peters MD Godfrey CM Khalil H McInerney P Parker D Soares CB. Guidance for conducting systematic scoping reviews. JBI Evid Implementation. (2015) 13(3):141–6. 10.1097/XEB.0000000000000050 26134548

[79] Tricco AC Lillie E Zarin W O'Brien KK Colquhoun H Levac D PRISMA Extension for scoping reviews (PRISMA-ScR): checklist and explanation. Ann Intern Med. (2018) 169(7):467-+. 10.7326/M18-0850 30178033

[80] Kleber M Wicki A Cheetham M. Strategien zur gemeinsamen entscheidungsfindung in der therapie bei patient_innen mit onkologischen Erkrankungen. Therapeutische Umschau. (2022) 79(8):401–8. 10.1024/0040-5930/a001381 36164740

[81] U.S. National Library of Medicine. ClinicalTrials.gov. U.S. National Institutes of Health. (2000). Available online at: http://clinicaltrials.gov/ (Accessed August 01, 2025).

[82] ProQuest. ProQuest. Available online at: https://www.proquest.com/ (Accessed August 01, 2025).

[83] Team TE. EndNote 20 ed. Philadeplhia, PA: Clarivate (2013).

[84] DistillerSR. DistillerSR. Version 2.35 ed. Ottawa: DistillerSR Inc (2023).

[85] Landis JR Koch GG. The measurement of observer agreement for categorical data. Biometrics. (1977) 33:159–74. 10.2307/2529310 843571

[86] Han PKJ Klein WMP Arora NK. Varieties of uncertainty in health care: a conceptual taxonomy. Med Decis Making. (2011) 31(6):828–38. 10.1177/0272989X10393976 22067431 PMC3146626

[87] Miller EM Porter JE Barbagallo MS. The experiences of health professionals, patients, and families with truth disclosure when breaking bad news in palliative care: a qualitative meta-synthesis. Palliat Support Care. (2022) 20(3):433–44. 10.1017/S1478951521001243 35713348

[88] Chu JT Wang MP Shen C Viswanath K Lam TH Chan SSC. How, when and why people seek health information online: qualitative study in Hong Kong. Interact J Med Res. (2017) 6(2):e7000. 10.2196/ijmr.7000 PMC5743920 29233802

[89] Josfeld L Keinki C Pammer C Zomorodbakhsch B Hübner J. Cancer patients’ perspective on shared decision-making and decision aids in oncology. J Cancer Res Clin. (2021) 147(6):1725–32. 10.1007/s00432-021-03579-6 PMC8076112 33682014

[90] Hesse BW Nelson DE Kreps GL Croyle RT Arora NK Rimer BK Trust and sources of health information—the impact of the internet and its implications for health care providers: findings from the first health information national trends survey. Arch Intern Med. (2005) 165(22):2618–24. 10.1001/archinte.165.22.2618 16344419

[91] Case DO Given LM. Looking for information: A survey of research on information seeking, needs, and behavior. (2016).

[92] Wilson TD. Models in information behaviour research. J Doc. (1999) 55(3):249–70. 10.1108/EUM0000000007145

[93] Chang DT Abouassaly R Lawrentschuk N. Quality of health information on the internet for urolithiasis on the google search engine. Adv Urol. (2016) 2016:8243095. 10.1155/2016/8243095 28044076 PMC5164884

[94] Suki NM. Handbook of Research on Leveraging Consumer Psychology for Effective Customer Engagement. Hershey: IGI Global (2016).

[95] Maddock C Lewis I Ahmad K. Sullivan R. Online information needs of cancer patients and their organizations. Ecancermedicalscience. (2011) 5:235. 10.3332/ecancer.2011.235 22276067 PMC3239170

[96] Kianifar F Pourhosseini SME Ansari Jaberi A Negahban Bonabi T. Cancer family members’ needs-based education in the management of anxiety: a randomized controlled clinical trial. Jundishapur J Chronic Dis Care. (2024) 13:e145119. 10.5812/jjcdc-145119

[97] Labaf A Jahanshir A Baradaran H Shahvaraninasab A. Is it appropriate to use western guidelines for breaking bad news in non-western emergency departments? A patients’ perspective. Clin Ethics. (2015) 10(1-2):13–21. 10.1177/1477750915581797

[98] Charlton RC. Breaking bad news. Med J Aust. (1992) 157(9):615–21. 10.5694/j.1326-5377.1992.tb137405.x 1406423

[99] Fallowfield L. Giving sad and bad news. Lancet. (1993) 341(8843):476–8. 10.1016/0140-6736(93)90219-7 8094499

[100] Buckman R. How to Break bad News: A Guide for Health Care Professionals. Baltimore, MA: University of Toronto Press (1992).

[101] Brewin TB. Three ways of giving bad news. Lancet. (1991) 337(8751):1207–9. 10.1016/0140-6736(91)92870-8 1673749

[102] Peteet JR Abrams HE Ross DM Stearns NM. Presenting a diagnosis of cancer: patients’ views. J Fam Pract. (1991) 32(6):577–81. 2040882

[103] Quill TE Townsend P. Bad news: delivery, dialogue, and dilemmas. Arch Intern Med. (1991) 151(3):463–8. 10.1001/archinte.1991.00400030033006 2001128

[104] Campbell ML. Breaking bad news to patients. JAMA. (1994) 271(13):1052. 10.1001/jama.271.13.1052 8139065

[105] Kaye P. Breaking Bad News: A ten Step Approach. Oxfordshire: Scion Publishing Ltd (2023).

[106] Vandekieft GK. Breaking bad news. Am Fam Physician. (2001) 64(12):1975–8. 11775763

[107] Abazari P Taleghani F Hematti S Ehsani M. Exploring perceptions and preferences of patients, families, physicians, and nurses regarding cancer disclosure: a descriptive qualitative study. Support Care Cancer. (2016) 24(11):4651–9. 10.1007/s00520-016-3308-x 27296237

[108] Randall TC Wearn AM. Receiving bad news: patients with haematological cancer reflect upon their experience. Palliative Med. (2005) 19(8):594–601. 10.1191/0269216305pm1080oa 16450876

[109] Tobin GA Begley C. Receiving bad news A phenomenological exploration of the lived experience of receiving a cancer diagnosis. Cancer Nurs. (2008) 31(5):E31–E9. 10.1097/01.NCC.0000305767.42475.7a 18772654

[110] Warnock C Tod A Foster J Soreny C. Breaking bad news in inpatient clinical settings: role of the nurse. J Adv Nurs. (2010) 66(7):1543–55. 10.1111/j.1365-2648.2010.05325.x 20492016

[111] Bousquet G Orri M Winterman S Brugière C Verneuil L Revah-Levy A. Breaking bad news in oncology: a metasynthesis. J Clin Oncol. (2015) 33(22):2437–U44. 10.1200/JCO.2014.59.6759 26124489

[112] Dewar A. Nurses’ experiences in giving bad news to patients with spinal cord injuries. J Neurosci Nurs. (2000) 32(6):324–30. 10.1097/01376517-200012000-00006 11155347

[113] Miyaji NT. The power of compassion: truth-telling among American doctors in the care of dying patients. Soc Sci Med. (1993) 36(3):249–64. 10.1016/0277-9536(93)90008-R 8426968

[114] Elit L Charles C Gafni A Ranford J Gold ST Gold I. Walking a tightrope: oncologists’ perspective on providing information to women with recurrent ovarian cancer (ROC) during the medical encounter. Support Care Cancer. (2012) 20:2327–33. 10.1007/s00520-011-1344-0 22167296

[115] Friedrichsen MJ Strang PM. Doctors’ strategies when breaking bad news to terminally ill patients. J Palliat Med. (2003) 6(4):565–74. 10.1089/109662103768253678 14516498

[116] Wenrich MD Curtis JR Shannon SE Carline JD Ambrozy DM Ramsey PG. Communicating with dying patients within the spectrum of medical care from terminal diagnosis to death. Arch Intern Med. (2001) 161(6):868–74. 10.1001/archinte.161.6.868 11268231

[117] Eggly S Penner L Albrecht TL Cline RJ Foster T Naughton M Discussing bad news in the outpatient oncology clinic: rethinking current communication guidelines. J Clin Oncol. (2006) 24(4):716–9. 10.1200/JCO.2005.03.0577 16446346

[118] Hack TF Degner LF Parker PA. The communication goals and needs of cancer patients: a review. Psycho-oncology: journal of the psychological. Soc Behav Dimens Cancer. (2005) 14(10):831–45. 10.1002/pon.949 16200519

[119] Matthews T Baken D Ross K Ogilvie E Kent L. The experiences of patients and their family members when receiving bad news about cancer: a qualitative meta-synthesis. Psycho-Oncology. (2019) 28(12):2286–94. 10.1002/pon.5241 31617646

[120] Ptacek J Ptacek JJ Ellison NM. “I'm sorry to tell you..” Physicians’ reports of breaking bad news. J Behav Med. (2001) 24:205–17. 10.1023/A:1010766732373 11392920

[121] Orlander JD Graeme Fincke B Hermanns D Johnson GA. Medical residents’ first clearly remembered experiences of giving bad news. J Gen Intern Med. (2002) 17(11):825–40. 10.1046/j.1525-1497.2002.10915.x 12406353 PMC1495131

[122] Salander P. Bad news from the patient’s perspective: an analysis of the written narratives of newly diagnosed cancer patients. Soc Sci Med. (2002) 55(5):721–32. 10.1016/S0277-9536(01)00198-8 12190266

[123] Eggly S Harper F Greene M. Companion Questions During Discussions of bad News in the Outpatient Oncology Setting. the 26th Annual Meeting of the Society of Behavioral Medicine. Boston, MA: Social Science & Medicine (2005).

[124] Fröjd C Lampic C Larsson G Birgegård G Lv E. Patient attitudes, behaviours, and other factors considered by doctors when estimating cancer patients’ anxiety and desire for information. Scand J Caring Sci. (2007) 21(4):523–9. 10.1111/j.1471-6712.2007.00507.x 18036016

[125] Wood LE. User Interface Design: Bridging the gap from User Requirements to Design. Boca Raton, FL: CRC Press (2018).

[126] Norman DA. The Psychology of Everyday Things. New York, NY: Basic books (1988).

[127] Gulliksen J Göransson B Boivie I Blomkvist S Persson J Cajander Å. Key principles for user-centred systems design. Behav Inform Technol. (2003) 22(6):397–409. 10.1080/01449290310001624329

[128] Dabbs ADV Myers BA Mc Curry KR Dunbar-Jacob J Hawkins RP Begey A User-centered design and interactive health technologies for patients. Compu Inform Nurs. (2009) 27(3):175–83. 10.1097/NCN.0b013e31819f7c7c PMC2818536 19411947

[129] Coulter A Stilwell D Kryworuchko J Mullen PD Ng CJ van der Weijden T. A systematic development process for patient decision aids. BMC Med Inform Decis Mak. (2013) 13 Suppl 2(Suppl 2):S2. 10.1186/1472-6947-13-S2-S2 24625093 PMC4044159

[130] Choe EK Duarte ME Suh H Pratt W Kientz JA. Communicating bad news: insights for the design of consumer health technologies. JMIR Human Factors. (2019) 6(2):e8885. 10.2196/humanfactors.8885 31102374 PMC6543800

[131] Horwitz AG Mills ED Sen S Bohnert AS. Comparative effectiveness of three digital interventions for adults seeking psychiatric services: a randomized clinical trial. JAMA Netw Open. (2024) 7(7):e2422115-e. 10.1001/jamanetworkopen.2024.22115 39023893 PMC11258584

[132] Sawesi S Rashrash M Phalakornkule K Carpenter JS Jones JF. The impact of information technology on patient engagement and health behavior change: a systematic review of the literature. JMIR Med Inform. (2016) 4(1):e4514. 10.2196/medinform.4514 PMC4742621 26795082

[133] Thodé M Pasman HRW Van Vliet LM Damman OC Ket JC Francke AL Feasibility and effectiveness of tools that support communication and decision making in life-prolonging treatments for patients in hospital: a systematic review. BMJ Support Palliat Care. (2022) 12(3):262–9. 10.1136/bmjspcare-2020-002284 PMC9411882 33020150

[134] Kilbride MK Joffe S. The new age of patient autonomy: implications for the patient-physician relationship. JAMA. (2018) 320(19):1973–4. 10.1001/jama.2018.14382 30326026 PMC6988779

[135] Stacey D Legare F Col NF Bennett CL Barry MJ Eden KB Decision aids for people facing health treatment or screening decisions. Cochrane Database Syst Rev. (2014) 1:CD001431. 10.1002/14651858.CD001431.pub5 24470076

[136] Golan T Purim O Rosin D Sapir E Gatt M Charas T Multi-institutional validation survey on belong. Life’s conversational artificial intelligence (AI) oncology mentor,” dave. J Clin Oncol. (2024) 42. 10.1200/JCO.2024.42.16_suppl.e1359639088774

[137] Belong.Life. BelongAI Dave—Cancer Mentor. (2025). Available online at: https://belong.life/press/belong-life-launches-dave-2/ (Accessed August 28, 2025).

[138] Světlák M. Virtual waiting room: the new narrative of waiting in oncology care. J Cancer Educ. (2025) 40(2):303–12. 10.1007/s13187-024-02496-9 39222293 PMC11978718

