# Supplementary material for: The waiting room of uncertainty: digital patient support for potentially bad news—a scoping review
Source: Front Digit Health. 2025 Nov 19;7:1706839. doi: 10.3389/fdgth.2025.1706839 (PMC12673405; doi:10.3389/fdgth.2025.1706839)
Supplement: Supplementary file 1 [file Datasheet1.pdf]

Supplementary Material

Supplemental Table 1. Summary of the qualitative systematic literature search, conducted across the eight electronic databases MEDLINE (Ovid), EMBASE (Ovid), Web of Science Core Collection, CINAHL, Cochrane Library (CENTRAL), IEEE Xplore Digital Library, PsycINFO, and Google Scholar. In addition, ClinicalTrials.gov was searched for ongoing trials and ProQuest for unpublished dissertations.

MEDLINE (Ovid)

	**Search terms**	**Limits (filter, limits, expander, refine)**
#1	("Digital Technology"[Mesh] OR "interactive Digital Technolog*"[tw] OR "Digital Technolog*"[tw] OR "Digital health"[tw] OR digital[tw] OR "internet-based"[tw] OR internet[tw] OR online[tw] OR computer[tw] OR "computer-based"[tw] OR "computer-assisted"[tw] OR electronic[tw] or web[tw] or "web-based"[tw] OR video[tw] OR "video conference"[tw] OR mobile[tw] OR smartphone[tw] OR phone[tw] OR handy[tw] OR tablet[tw] OR iPad[tw] OR android[tw] or software[tw] OR notebook[tw] OR laptop[tw] OR mobile[tw] OR smartphone[tw] OR handheld[tw] OR audio[tw] OR media[tw] OR medien[tw] OR virtual[tw] OR augmented[tw] OR interactive[tw] OR "mobile applications"[tw] OR "digital support*"[tw] OR "digital aid*"[tw] OR "digital tool*"[tw] or "digital instrument*"[tw] OR "digital technique*"[tw] OR "digital system*"[tw] OR "digital program*"[tw] OR "digital algorithm*"[tw] OR "digital process*"[tw] OR "digital method*"[tw] OR "digital intervention*"[tw] OR "digital material*"[tw] OR "digital guidance*"[tw] OR "interactive health communication"[tw] OR "interactive internet"[tw] OR "interactive online"[tw] OR "interactive graphic"[tw] OR "interactive booklet"[tw] OR "interactive tool*"[tw] OR "communication package"[tw] OR "digital health communication"[tw] OR "interactive health information"[tw] OR "interactive health communication"[tw] OR "interactive health education"[tw] OR "patient guidance system"[tw])	
#2	("Neoplasms"[Mesh] OR "Medical Oncology"[Mesh] OR oncolog*[tw] OR neoplas*[tw] OR malignanc*[tw] OR tumor*[tw] OR tumour*[tw])	

#3	("breaking bad news"[tw] OR "receiving bad news"[tw] OR "Truth Disclosure"[Mesh] OR "bad news"[tw] OR "communicating bad news"[tw] OR "delivering bad news"[tw])	
#4	#1 AND #2 AND #3	
#5	#1 AND #2 AND #3	English language
#6	("Decision Making, Shared"[Mesh] OR "shared decision making"[tw] OR "shared decision"[tw] OR decision*[tw] OR "patient decision making"[tw] OR "informed consent"[tw] OR "informed choice"[tw] OR "informed decision"[tw] OR "informed decision"[tw] OR "decision making"[tw] OR "choice behavior"[tw] OR "choice behaviour"[tw])	
#7	#4 AND #6	
#8	#4 AND #6	English language

Web of Science Core Collection

	Search terms	Limits (filter, limits, expander, refine)
#1	TS=("interactive digital technolog*" OR "digital technolog*" OR "digital health" OR digital OR "internet-based" OR internet OR online OR computer OR "computer-based" OR "computer-assisted" OR electronic OR web OR "web-based" OR video OR "video conference" OR phone OR handy OR tablet OR ipad OR android OR software OR notebook OR laptop OR mobile OR smartphone OR handheld OR audio OR media OR medien OR virtual OR augmented OR interactive OR "mobile applications" OR "digital support*" OR "digital aid*" OR "digital tool*" OR "digital instrument*" OR "digital technique*" OR "digital system*" OR "digital program*" OR "digital algorithm*" OR "digital process*" OR "digital method*" OR "digital intervention*" OR "digital material*" OR "digital guidance*" OR "interactive internet" OR "interactive online" OR "interactive graphic" OR "interactive booklet" OR "interactive tool*" OR "communication package" OR "digital health communication" OR "interactive health information" OR "interactive health communication" OR "interactive health education" OR "patient guidance system")	
#2	TS=("breaking bad news" OR (breaking NEAR/3 "bad news") OR "receiving bad news" OR (receiving NEAR/3 "bad news") OR "truth disclosure" OR "bad news" OR "communicating bad news" OR "delivering bad news")	
#3	TS=(*oncolog* OR neoplas* OR malignan* OR tumo$r*)	
#4	#1 AND #2 AND #3	
#5	#1 AND #2 AND #3	English
#6	TS=((shared NEAR/3 "decision making") OR (patient NEAR/3 "decision making") OR "shared decision making" OR "shared decision" OR decision* OR "patient decision making" OR "informed consent" OR "informed choice" OR "informed decision" OR "decision making" OR "choice behavior" OR "choice behavio$r")	
#7	#4 AND #6	

	**Search terms**	**Limits (filter, limits, expander, refine)**
#8	#4 AND #6	English

CINAHL (Database search of CINAHL via EBSCO Host)

	**Search terms**	**Limits (filter, limits, expanders, refine**
#1	((MH "Digital Technology+") OR (MH "Digital Health+") OR “interactive digital technolog*” OR “digital technolog*” OR "software" OR “internet-based” OR "phone" OR "laptop" OR “digital health” OR digital OR internet OR online OR computer OR “computer-based” OR “computer-assisted” OR electronic OR web OR “web-based” OR video OR “video conference” OR handy OR tablet OR ipad OR android OR notebook OR mobile OR smartphone OR handheld OR audio OR media OR medien OR virtual OR augmented OR interactive OR “mobile applications” OR “digital support*” OR “digital aid*” OR “digital tool*” OR “digital instrument*” OR “digital technique*” OR “digital system*” OR “digital program*” OR “digital algorithm*” OR “digital process*” OR “digital method*” OR “digital intervention*” OR “digital material*” OR “digital guidance*” OR “interactive internet” OR “interactive online” OR “interactive graphic” OR “interactive booklet” OR “interactive tool*” OR “communication package” OR “digital health communication” OR “interactive health information” OR “interactive health communication” OR “interactive health education” OR “patient guidance system”)	All fields
#2	(“breaking bad news” OR “receiving bad news” OR (MH "Truth Disclosure+") OR "bad news" OR “delivering bad news” OR “communicating bad news”)	All fields
#3	((MH "Oncologic Care+") OR (MH "Oncology+") OR "*oncolog*" OR (MH "Neoplasms+") OR "malign*" OR "tumo#r*" OR "neoplasm*" OR “cancer*”)	All fields
#4	#1 AND #2 AND #3	All fields
#5	#1 AND #2 AND #3	All fields<br>Language: English

#6	((MH "Decision Making, Shared") OR (MH "Decision Making+") OR “shared decision” OR (MH "Decision Making, Patient+") OR “shared decision making” OR “patient decision making” OR (MH "Consent+") OR “informed consent” OR “informed choice” OR “informed decision” OR (MH "Decision Making, Computer Assisted+") OR “decision making” OR “choice behavio#r” OR “decision”)	All fields
#7	#5 AND #6	All fields
#8	#6 AND #7	All fields<br>Language: English

Cochrane Library (CENTRAL: Cochrane Central Register of Controlled Trials)

	Search terms	Limits (filter, limits, expanders, refine)
**#1**	MeSH descriptor: [Digital Technology] explode all trees	MESH
**#2**	("interactive Digital Technolog*":ti,ab,kw OR "Digital NEAR Technolog*":ti,ab,kw OR "Digital health":ti,ab,kw OR digital:ti,ab,kw OR "internet-based":ti,ab,kw OR internet:ti,ab,kw OR online:ti,ab,kw OR computer:ti,ab,kw OR "computer-based":ti,ab,kw OR "computer-assisted":ti,ab,kw OR electronic:ti,ab,kw or web:ti,ab,kw or "web-based":ti,ab,kw OR video:ti,ab,kw OR "video conference":ti,ab,kw OR mobile:ti,ab,kw OR smartphone:ti,ab,kw OR phone:ti,ab,kw OR handy:ti,ab,kw OR tablet:ti,ab,kw OR iPad:ti,ab,kw OR android:ti,ab,kw or software:ti,ab,kw OR notebook:ti,ab,kw OR laptop:ti,ab,kw OR mobile:ti,ab,kw OR smartphone:ti,ab,kw OR handheld:ti,ab,kw OR audio:ti,ab,kw OR media:ti,ab,kw OR medien:ti,ab,kw OR virtual:ti,ab,kw OR augmented:ti,ab,kw OR interactive:ti,ab,kw OR "mobile applications":ti,ab,kw OR "digital support*":ti,ab,kw OR "digital aid*":ti,ab,kw OR "digital tool*":ti,ab,kw or "digital instrument*":ti,ab,kw OR "digital technique*":ti,ab,kw OR "digital system*":ti,ab,kw OR "digital program*":ti,ab,kw OR "digital algorithm*":ti,ab,kw OR "digital process*":ti,ab,kw OR "digital method*":ti,ab,kw OR "digital intervention*":ti,ab,kw OR "digital material*":ti,ab,kw OR "digital guidance*":ti,ab,kw OR "interactive health communication":ti,ab,kw OR "interactive internet":ti,ab,kw OR "interactive online":ti,ab,kw OR "interactive graphic":ti,ab,kw OR "interactive booklet":ti,ab,kw OR "interactive tool*":ti,ab,kw OR "communication package":ti,ab,kw OR "digital health communication":ti,ab,kw OR "interactive health information":ti,ab,kw OR "interactive health communication":ti,ab,kw OR "interactive health education":ti,ab,kw OR "patient guidance system":ti,ab,kw)	
**#3**	MeSH descriptor: [Internet] explode all trees	
**#4**	MeSH descriptor: [Computers] explode all trees	
**#5**	(#1 OR #2 OR #3 OR #4)	

**#6**	MeSH descriptor: [Medical Oncology] explode all trees	
**#7**	(oncolog*:ti,ab,kw OR neoplas*:ti,ab,kw OR malignanc*:ti,ab,kw OR tumor*:ti,ab,kw OR tumour*:ti,ab,kw)	
**#8**	MeSH descriptor: [Neoplasms] explode all trees	
**#9**	(#6 OR #7 OR #8)	
**#10**	MeSH descriptor: [Truth Disclosure] explode all trees	
**#11**	("breaking bad news":ti,ab,kw OR "receiving bad news":ti,ab,kw OR "bad news":ti,ab,kw OR "communicating bad news":ti,ab,kw OR "delivering bad news":ti,ab,kw)	
**#12**	(#10 OR #11)	
**#13**	#5 AND #9 AND #12	
**#14**	#5 AND #9 AND #12	English
**#15**	MeSH descriptor: [Decision Making, Shared] explode all trees	
**#16**	MeSH descriptor: [Informed Consent] explode all trees	
**#17**	("shared decision making":ti,ab,kw OR "shared NEAR (decision making)":ti,ab,kw OR "shared decision":ti,ab,kw OR decision*:ti,ab,kw OR “patient decision making”:ti,ab,kw OR “informed consent”:ti,ab,kw OR “informed choice”:ti,ab,kw OR “informed decision”:ti,ab,kw OR “informed decision”:ti,ab,kw OR “decision making”:ti,ab,kw OR “choice behavior”:ti,ab,kw)	
**#18**	#15 OR #16 OR #17	
**#19**	(#13 AND #18)	
**#20**	(#13 AND #18)	English

IEEE Xplore Digital Library

	Search terms	Limits (filter, limits, expander, refine)
**#1**	"interactive Digital Technolog*" OR "Digital Technolog*" OR "Digital health" OR digital OR "internet-based" OR internet OR online OR computer OR "computer-based" OR "computer-assisted" OR electronic or web or "web-based" OR video OR "video conference" OR mobile OR smartphone OR phone OR handy OR tablet OR iPad OR android or software OR notebook OR laptop OR mobile OR smartphone OR handheld OR audio OR media OR medien OR virtual OR augmented OR interactive OR "mobile applications" OR "digital support" OR "digital supports" OR "digital aid" OR "digital aids" OR "digital tool" OR "digital tools" OR "digital instrument" OR "digital instruments" OR "digital technique" OR "digital system*" OR "digital program*" OR "digital algorithm*" OR "digital process*" OR "digital method" OR "digital methods" OR "digital intervention*" OR "digital material*" OR "digital guidance" OR "interactive health communication" OR "interactive internet" OR "interactive online" OR "interactive graphic" OR "interactive booklet" OR "interactive tool" OR "interactive tools" OR "communication package" OR "digital health communication" OR "interactive health information" OR "interactive health communication" OR "interactive health education" OR "patient guidance system"	all metadata
**#2**	"medical oncology" OR oncolog* OR neoplas* OR malignanc* OR tumor* OR tumour*	all metadata
**#3**	"breaking bad news" OR breaking NEAR/3 “bad news” OR receiving NEAR/3 “bad news” OR "receiving bad news" OR "Truth Disclosure" OR "bad news" OR "communicating bad news" OR "delivering bad news"	all metadata
**#4**	#1 AND #2 AND #3	all metadata
**#5**	#1 AND #2 AND #3	all metadata English

**#6**	"shared decision making" OR shared NEAR/3 “decision making” OR "shared decision" OR decision* OR "patient decision making" OR patient NEAR/3 “decision making” OR "informed consent" OR "informed choice" OR "informed decision*" OR "decision making" OR "choice behavior" OR "choice behaviour"	all metadata
**#7**	#1 AND #2 AND #3 AND #4	all metadata
**#8**	#1 AND #2 AND #3 AND #4	all metadata English

PsycINFO (Search of database via EBSCO Host)

	**Search terms**	**Limits (filter, limits, expanders, refine)**
#1	(DE "Digital Technology" OR DE "Health Information" OR DE "Digital Information" OR “interactive digital technolog*” OR “digital technolog*” OR "software" OR “internet-based” OR "phone" OR "laptop" OR “digital health” OR digital OR internet OR online OR computer OR “computer-based” OR “computer-assisted” OR electronic OR web OR “web-based” OR video OR “video conference” OR handy OR tablet OR ipad OR android OR notebook OR mobile OR smartphone OR handheld OR audio OR media OR medien OR virtual OR augmented OR interactive OR “mobile applications” OR “digital support*” OR “digital aid*” OR “digital tool*” OR “digital instrument*” OR “digital technique*” OR “digital system*” OR “digital program*” OR “digital algorithm*” OR “digital process*” OR “digital method*” OR “digital intervention*” OR “digital material*” OR “digital guidance*” OR “interactive internet” OR “interactive online” OR “interactive graphic” OR “interactive booklet” OR “interactive tool*” OR “communication package” OR “digital health communication” OR “interactive health information” OR “interactive health communication” OR “interactive health education” OR “patient guidance system”)	All fields
#2	(“breaking bad news” OR “receiving bad news” OR "Truth Disclosure” OR "bad news" OR “delivering bad news” OR "communicating bad news")	All fields
#3	(DE "Neoplasms" OR DE "Oncology" OR "*oncolog*" OR "malign*" OR tumor* OR tumour* OR neoplasm OR “cancer*”)	All fields
#4	(DE "Digital Technology" OR DE "Health Information" OR DE "Digital Information" OR “interactive digital technolog*” OR “digital technolog*” OR "software" OR “internet-based” OR "phone" OR "laptop" OR “digital health” OR digital OR internet OR online OR computer OR “computer-based” OR “computer-assisted” OR electronic OR web OR “web-based” OR video OR “video conference” OR handy OR tablet OR ipad OR android OR notebook OR mobile OR smartphone OR handheld OR audio OR media OR medien OR virtual OR augmented OR interactive OR “mobile applications” OR “digital support*” OR “digital aid*” OR “digital tool*” OR “digital instrument*” OR “digital technique*” OR “digital system*” OR “digital program*” OR “digital algorithm*” OR “digital process*” OR “digital method*” OR “digital intervention*” OR “digital material*” OR “digital guidance*” OR “interactive internet” OR “interactive online” OR “interactive graphic” OR “interactive booklet” OR “interactive tool*” OR “communication package” OR “digital health communication” OR “interactive health information” OR	All fields

	“interactive health communication” OR “interactive health education” OR “patient guidance system”) AND (“breaking bad news” OR “receiving bad news” OR "Truth Disclosure” OR "bad news" OR “delivering bad news” OR "communicating bad news") AND (DE "Neoplasms" OR DE "Oncology" OR "*oncolog*" OR "malign*" OR tumor* OR tumour* OR neoplasm OR “cancer*”)	
#5	(DE "Digital Technology" OR DE "Health Information" OR DE "Digital Information" OR “interactive digital technolog*” OR “digital technolog*” OR "software" OR “internet-based” OR "phone" OR "laptop" OR “digital health” OR digital OR internet OR online OR computer OR “computer-based” OR “computer-assisted” OR electronic OR web OR “web-based” OR video OR “video conference” OR handy OR tablet OR ipad OR android OR notebook OR mobile OR smartphone OR handheld OR audio OR media OR medien OR virtual OR augmented OR interactive OR “mobile applications” OR “digital support*” OR “digital aid*” OR “digital tool*” OR “digital instrument*” OR “digital technique*” OR “digital system*” OR “digital program*” OR “digital algorithm*” OR “digital process*” OR “digital method*” OR “digital intervention*” OR “digital material*” OR “digital guidance*” OR “interactive internet” OR “interactive online” OR “interactive graphic” OR “interactive booklet” OR “interactive tool*” OR “communication package” OR “digital health communication” OR “interactive health information” OR “interactive health communication” OR “interactive health education” OR “patient guidance system”) AND (“breaking bad news” OR “receiving bad news” OR "Truth Disclosure” OR "bad news" OR “delivering bad news” OR "communicating bad news") AND (DE "Neoplasms" OR DE "Oncology" OR "*oncolog*" OR "malign*" OR tumor* OR tumour* OR neoplasm OR “cancer*”)	All fields Language: English
#6	(DE "Decision Making" OR DE "Informed Consent" OR “shared decision” “shared decision making” OR “patient decision making” OR “informed consent” OR “informed choice” OR “informed decision” OR “decision making” OR “choice behaviour” OR "choice behavior" OR “decision”)	All fields
#7	(DE "Digital Technology" OR DE "Health Information" OR DE "Digital Information" OR “interactive digital technolog*” OR “digital technolog*” OR "software" OR “internet-based” OR "phone" OR "laptop" OR “digital health” OR digital OR internet OR online OR computer OR “computer-based” OR “computer-assisted” OR electronic OR web OR “web-based” OR video OR “video conference” OR handy OR tablet OR ipad OR android OR notebook OR mobile OR smartphone OR handheld OR audio OR media OR medien OR virtual OR augmented OR interactive OR “mobile applications” OR “digital support*” OR “digital aid*” OR “digital tool*” OR “digital instrument*” OR “digital technique*” OR “digital system*” OR “digital program*” OR “digital algorithm*” OR “digital process*” OR “digital method*” OR “digital intervention*” OR “digital material*” OR “digital guidance*” OR “interactive internet” OR “interactive online” OR “interactive graphic” OR “interactive booklet” OR “interactive tool*” OR “communication package” OR “digital health communication” OR “interactive health information” OR	All fields

	“interactive health communication” OR “interactive health education” OR “patient guidance system”) AND (“breaking bad news” OR “receiving bad news” OR "Truth Disclosure” OR "bad news" OR “delivering bad news” OR "communicating bad news") AND (DE "Neoplasms" OR DE "Oncology" OR "*oncolog*" OR "malign*" OR tumor* OR tumour* OR neoplasm OR “cancer*”) AND (DE "Decision Making" OR DE "Informed Consent" OR “shared decision” “shared decision making” OR “patient decision making” OR “informed consent” OR “informed choice” OR “informed decision” OR “decision making” OR “choice behaviour” OR "choice behavior" OR “decision”)	
#8	(DE "Digital Technology" OR DE "Health Information" OR DE "Digital Information" OR “interactive digital technolog*” OR “digital technolog*” OR "software" OR “internet-based” OR "phone" OR "laptop" OR “digital health” OR digital OR internet OR online OR computer OR “computer-based” OR “computer-assisted” OR electronic OR web OR “web-based” OR video OR “video conference” OR handy OR tablet OR ipad OR android OR notebook OR mobile OR smartphone OR handheld OR audio OR media OR medien OR virtual OR augmented OR interactive OR “mobile applications” OR “digital support*” OR “digital aid*” OR “digital tool*” OR “digital instrument*” OR “digital technique*” OR “digital system*” OR “digital program*” OR “digital algorithm*” OR “digital process*” OR “digital method*” OR “digital intervention*” OR “digital material*” OR “digital guidance*” OR “interactive internet” OR “interactive online” OR “interactive graphic” OR “interactive booklet” OR “interactive tool*” OR “communication package” OR “digital health communication” OR “interactive health information” OR “interactive health communication” OR “interactive health education” OR “patient guidance system”) AND (“breaking bad news” OR “receiving bad news” OR "Truth Disclosure” OR "bad news" OR “delivering bad news” OR "communicating bad news") AND (DE "Neoplasms" OR DE "Oncology" OR "*oncolog*" OR "malign*" OR tumor* OR tumour* OR neoplasm OR “cancer*”) AND (DE "Decision Making" OR DE "Informed Consent" OR “shared decision” “shared decision making” OR “patient decision making” OR “informed consent” OR “informed choice” OR “informed decision” OR “decision making” OR “choice behaviour” OR "choice behavior" OR “decision”)	All fields<br>Language: English

Google Scholar

	Search terms	Limits (filter, limits, expanders, refine)
#1	Oncology "interactive digital technology" "breaking bad news"	Language: English
#2	Oncology "digital technology" "breaking bad news"	Language: English
#3	Oncology "digital technology" "receiving bad news" "breaking bad news"	Language: English
#4	"Breaking bad news" "digital technology" oncology "shared decision making"	Language: English

http://clinicaltrials.gov/

	Search terms	Limits (filter, limits, refine)
#1	“Breaking bad news”	Study status: completed<br>Study results: with results
#2	“Receiving bad news”	Study status: completed<br>Study results: with results
#3	"Truth disclosure"	Study status: completed<br>Study results: with results
#4	“Delivering bad news"	Study status: completed<br>Study results: with results

ProQuest - Dissertations

	Search terms	Limits (filter, limits, refine)
#1	"Breaking bad news" AND oncology AND "digital technology"	Dissertation and Thesis A&I, "Anywhere except full text" for citations or abstracts
#2	"Receiving bad news" AND oncology AND "digital technology"	Dissertation and Thesis A&I, "Anywhere except full text" for citations or abstracts
#3	"Truth disclosure" AND oncology AND "digital technology"	Dissertation and Thesis A&I, "Anywhere except full text" for citations or abstracts

Supplemental Table 2. Summary of screening outcomes, listing the study citations, their categorization at the first screening step (title and abstract review), and the reasons for exclusion at the second screening step (full-text review).

	**Citation**	**Step 1 Categorization**	**Reason for Exclusion at Full-Text Screening**
1	Feldman H, Rodriguez ES. The future of oncology care with personal health records. American Society of Clinical Oncology Educational Book. 2012;32(1):e66-9.	Included	Related to telehealth
2	McNeil CM, Harnett PR. Our Faustian pact with the digital world. The Lancet Oncology. 2017 Feb 1;18(2):171-2.	Included	Comment
3	Debes JD. Virtual empathy and liver cancer. Liver International. 2020 Oct;40(10):2571-2571.	Unclear	Related to telehealth
4	Sajnic A, Karabatic S, Milicevic J, Belina I, Dodlek N, Jakopovic M. P2. 04-01 Lung Cancer Patient Experience Survey from Twelfth Central and Eastern Europe Countries. Journal of thoracic oncology. 2022 Sep 1;17(9):S132.	Unclear	Conference Poster
5	Richards HL, Fortune DG, Lyons L, Curtain Y, Hennessey D. "You happy enough with it doctor?": an exploratory qualitative examination of the communication that takes place during surveillance cystoscopy for non-muscle invasive bladder cancer. European Urology Open Science. 2022 Sep 1;43:S13.	Unclear	Conference Poster
6	Karabatić S, Šajnić A, Pleština S, Jakopović M, Kurtović B. Croatian national cancer patient experience survey. International journal of environmental research and public health. 2022 Jul 7;19(14):8285.	Unclear	Not about patient aids for BBN/RBN (it does include patient experience of BBN)
7	Kumar D, Gordon NP, Neeman E, Zamani C, Sheehan TR, Martin E, Payne J, Egorova O, Kolevska T, Liu R. Patient preferences for telehealth versus in-person oncology visits. *Journal of Clinical Oncology*, 2022; 40(28).	Included	Related to telehealth
8	Granberg RE, Heyer A, Rising KL, Handley NR, Gentsch AT, Binder AF. Medical oncology patient perceptions of telehealth video visits. JCO oncology practice. 2021 Sep;17(9):e1333-43.	Included	Related to telehealth
9	Malmström A, Åkesson L, Milos P, Mudaisi M, Bruhn H, Strandeus M, Karlsson M. "Do I want to know it all?" A qualitative study of glioma patients' perspectives on receiving information about their diagnosis and prognosis. Supportive Care in Cancer. 2021 Jun;29(6):3339-46.	Included	Conference Paper
10	Ahmad, H. and Van Staden, B. Use of pre-recorded audio-visual information for patients diagnosed with six common malignant haematological disorders with an aim to improve	Included	Conference Poster

	patient experience and facilitate breaking bad news, *Haematologica*, 2016 Jun 1; 101, p. 599-599.		
11	Hillard, J.R. Content analysis of postings in a stomach cancer survivor and caregiver support group on Facebook, *Journal of Clinical Oncology*, 2015; 33(3).	Included	Conference Paper
12	Han Y, Ravella H. Smart Agents at Your Service: Potential and Pitfalls. Advances in Consumer Research. 2021;49:768-73.	Included	Conference Paper (about humanoid service robots in clinical interaction).
13	Vasey J, Smith J, Mitchell N. Involving children, young people and families in care and care decisions. In Essentials of Nursing Children and Young People 2018 Jan 1 (pp. 3-19). SAGE Publications.	Unclear	Book chapter
14	Cross P. CA, Malkin A., Burrell H. et al. AB25 2ZD. SCAN: Scottish Cytopathology and Analytical Newsletter, 2019;30:2. www.britishcytology.org.uk/uploads/files/eSCAN_30-2_Oct_2019.pdf	Unclear	Professional magazine
15	Taylor M. Bibliography issue. Public Relations Review. 2007 Dec 1;33:445-532.	Unclear	Not enough information
16	Nicholson, C. Research roundup, *International Journal of Palliative Nursing*, 2014; 20(7), 359–360.	Unclear	Not about patient aids for BBN/RBN. Paper contains synopses of research articles in palliative care.
17	Abstracts: Oral presentations, *DYNAMICS*, The Official Journal of the Canadian Association of Critical Care Nurses, 2009; 20(2), 11–40.	Unclear	Not enough information (publication not identified)
18	Koh JJ. Why did my doctor call? Patient Education and Counseling. 2018 1;101(8):1506-7.	Unclear	Case report
19	Aschwanden, C. The many ways to share bad news, 2014; E1–E4.	Unclear	Not enough information
20	World in brief. Nursing Standard. 2013(47).	Unclear	Not enough information (publication not identified)
21	Andersen IC, Agerskov H, Kaasgaard M, Simonÿ C, Bodtger U. Receiving Bad News by Telephone: The Lived Experience of Patients Undergoing the Diagnostic Workup for a Suspected Lung Cancer Diagnosis. Cancer Nursing. 2025 Sep 8:10-97.	Included	Related to telehealth
22	Salmi, L., Hubbard, J. and McFarland, D.C. (2024) 'When bad news comes through the portal: Strengthening trust and guiding patients when they receive bad results before their clinicians', *American Society of Clinical Oncology Educational Book*, 44(3), e433944.	Unclear	Related to telehealth
